# TMEM119-positive microglial cells in cerebrospinal fluid, a potential new marker for neuroinflammatory response after aneurysmal subarachnoid hemorrhage

**DOI:** 10.1007/s00702-025-02886-3

**Published:** 2025-02-04

**Authors:** Andrea Cattaneo, Julia Messinger, Kevin Lamllari, Helmut Heinsen, Michael K. Schuhmann, Christoph Wipplinger, Vera Nickl, Mario Löhr, Ekkehard Kunze, Christian Stetter, Thomas Linsenmann, Michael Bohnert, Ralf-Ingo Ernestus, Johann Zwirner, Benjamin Ondruschka, Camelia-Maria Monoranu, Simone Bohnert

**Affiliations:** 1 Department of Neurosurgery, University Hospital of Würzburg, Würzburg, Germany; 2 Institute of Forensic Medicine, University of Würzburg, Würzburg, Germany; 3 Department of Neurology, University Hospital Würzburg, Würzburg, Germany; 4 Department of Neurologic Surgery, Mayo Clinic, Rochester, MN USA; 5 Institute of Legal Medicine, University Medical Center Hamburg-Eppendorf, Hamburg, Germany; 6 Department of Neuropathology, Institute of Pathology, University of Würzburg, Würzburg, Germany

**Keywords:** Subarachnoid hemorrhage, Microglia, Cerebrospinal fluid, Inflammation, TMEM119

## Abstract

Aneurysmal subarachnoid hemorrhage (aSAH) is a debilitating condition with significant morbidity and mortality rates. Despite advancements in treatment, understanding the underlying pathophysiology, particularly the inflammatory response, remains crucial for improving patient outcomes. In this study, we investigated the presence of transmembrane protein 119 (TMEM119) of microglial cells in cerebrospinal fluid (CSF) as a potential marker for neuroinflammation following aSAH. CSF samples were collected from aSAH patients, pathological and healthy controls, processed, and analyzed using immunocytochemistry. TMEM119-positive microglial cells were consistently identified in the CSF of aSAH patients, exhibiting amoeboid morphology and intense staining. Importantly, microglial cells were detected as early as the first day post-bleeding, persisting throughout the acute phase in some cases. Analysis of consecutive samples revealed varying trends in microglial cell numbers, with a peak during the initial phase followed by a gradual decline. Our findings suggest that microglia may migrate into the CSF following aSAH, potentially serving as an early predictor of inflammatory-related CNS damage. This study underscores the importance of understanding neuroinflammatory processes in aSAH and opens avenues for further research on the role of microglia in CNS disorders by liquid biopsy.

## Introduction

Aneurysmal subarachnoid hemorrhage (aSAH) represents a serious condition with high morbidity and mortality. Up to 45% of patients die in the first few months after bleeding, and a significant number of patients who survive must cope with significant disability and neurological deficits (Hoh et al. 2023; Wahood et al. 2022). A large part of the long-term deficits of patients with aSAH is due to the complications that occur in the subacute and chronic phase after bleeding (Etminan et al. 2019; Geraghty and Testai 2017). The pathophysiology of posthemorrhagic brain damage caused by aSAH is complex and still not completely understood (Geraghty and Testai 2017; Osgood 2021). Delayed cerebral ischemia (DCI) is one of the most common and important intracranial complications following the initial hemorrhage, contributing substantially to the overall disease burden (Geraghty and Testai 2017). While vasospasm has long been considered a primary cause of DCI and consequently of posthemorrhagic brain damage, it has become increasingly evident that microenvironment events such as microthrombosis, microvasospasm, spreading depolarizations, apoptosis/necrosis, disruption of the blood-brain barrier (BBB) and inflammatory processes within the central nervous system (CNS) also play a pivotal role in the development and progression of this damage (Patsouris et al. 2023; Mohme et al. 2020; Group et al. 2019; Roa et al. 2020; Alsbrook et al. 2023; Liao et al. 2023). The neuroinflammatory response in aSAH is a complex and multifaceted process, it is triggered immediately after blood extravasation and it evolves into a structured reaction in the chronic phase (Alsbrook et al. 2023; Weiland et al. 2021). Immediately after blood extravasation, the parenchyma and the subarachnoid space are invaded by immune cells, both attracted and activated by the inflammatory environment created and facilitated by the destruction of the blood-brain barrier (Li et al. 2022). The neuroinflammatory response is now recognized as a significant component of the pathophysiology of aSAH (Osgood 2021; Mohme et al. 2020; Romoli et al. 2023). One of the most important cell populations involved in the neuroinflammatory response is microglia, the resident macrophage of the CNS (Prinz et al. 2019). Microglia is known to rapidly respond to various pathological insults, becoming activated with a different polarization and contributing to both neuroprotection, neuroinflammation and neurodegeneration (Prinz et al. 2019; Böttcher et al. 2019; Sankowski et al. 2029). The role of microglia in initiating and perpetuating inflammation in the context of aSAH has gained increasing attention (Schneider et al. 2015; Chen et al. 2022; Zeng et al. 2020). Even though the precise role of microglia in aSAH is not fully defined, there are data from animal studies indicating that microglia play a key role in sustaining damage after bleeding (Schneider et al. 2015). These cells may serve as a valuable indicator of the inflammatory state of the brain following aSAH (Zeng et al. 2020).While inflammatory events are challenging to be studied directly in brain parenchyma due to limited accessibility, the presence and dynamics of microglial cells in the cerebrospinal fluid (CSF) may offer an alternative route to investigate the extent and development of neuroinflammation in aSAH patients. Furthermore, it holds the promise of becoming a potential prognostic marker, helping clinicians predict the risk of DCI and poor clinical outcomes. To our knowledge the only description of microglial cells in CSF after aSAH was made with flow cytometry (Roa et al. 2020).

This study aims to confirm immunocytochemically the presence of microglial cells in CSF using TMEM119, a trans-membranous molecule which was proved to be a specific and robust microglial marker even though it can be downregulated in some contexts (Satoh et al. 2016; Mercurio et al. 2022; Ruan and Elyaman 2022; Ruan et al. 2020; Vankriekelsvenne et al. 2022; Bobotis et al. 2024). Furthermore, we aim to validate the data previously described by our group (Bohnert et al. 2020, 2022) in post-mortem specimens by now conducting our studies in living patients.

## Materials and methods

### Cerebrospinal fluid (CSF) collection and processing

CSF samples for aSAH patients and pathological controls were obtained via external ventricular drainage and in two cases via lumbar drainage. All the patients and pathological controls were treated in the intensive care unit of the Department of Neurosurgery of the University Hospital Würzburg. The collection process involved discarding the initial 2–5 ml of CSF during routine morning drainage for all included patients. All samples were promptly transported to our laboratory for immediate analysis, as part of our standard practice for daily CSF assessments in our institution. Upon arrival, the remaining CSF was centrifuged at 5000 rpm for 5 min at 4 °C to produce cytospin preparations, as described in precedent publications of our group (Bohnert et al. 2019, 2022). These slides were sent to the Institute of Forensic Medicine of the university of Würzburg for staining, ensuring the anonymization of the material. For healthy control, CSF cytospin samples were acquired from three anonymized patients (age-matched) without underlying pathology from the Institute of Pathology. All patients in the healthy control group underwent a lumbar puncture for suspected neoplastic disease (various types of lymphoma) but were not diagnosed with central nervous system pathology. The selected samples from the healthy control group were cytospins, processed in line with other samples, and subjected to identical centrifugation and staining preparation procedures.

### Patient samples and data collection

Samples were obtained from 20 patients with external ventricular drainage and 3 healthy controls between February 2023 and June 2023 at our institution. Clinical data were collected and stored in an anonymized database using Excel (Microsoft Corporation, Redmond, USA).

### Staining and immunocytochemistry

Cytospin preparations were mounted on microscope slides and subjected to Mayer’s hematoxylin-eosin (HE) staining and immunocytochemical processing. Commercially available antibodies against TMEM119 (1:1000 dilution, HP A051870 Sigma, St. Louis, USA) and CD68 (1:100 dilution, 168 M-9, Sigma, St. Louis, USA) were used as primary antibodies, following established protocols from our previous studies (Bohnert et al. 2022; Bohnert et al. 2019). The MultiLink Streptavidin-Peroxidase-Kit (BioGenex, San Ramon, USA) served as the secondary antibody. Control slides were included to detect any nonspecific staining, achieved by omitting the primary antibodies during staining procedures.

### Imaging and analysis

Microscopic digital images of the stained cytospin preparations were captured using a Leica digital camera DMC 5400 mounted on a Leica DM6 B microscope at a consistent magnification of 100x (both Leica Microsystems Corporation, Wetzlar, Germany). Five images were systematically taken from each cytospin preparation in a standardized manner, ensuring uniform representation across different areas of the slide. The five images per cytospin were taken in a clockwise manner at 12, 3, 6, and 9 o’clock together with the center of each circular cytospin preparation as described before (Bohnert et al. 2022; Bohnert et al. 2019).

For quantitative assessment, image processing software (Leica LASX, Wetzlar, Germany) was utilized, as previously described. Parameters defining cell morphology and staining intensity for the TMEM119 and CD68 antibody were established and consistently applied for digital image analysis. The software automatically compiled data into an Excel table, measuring the number of TMEM119 and CD68 positive cells per field of view. The number of cells were then added together as a total.

### Ethics approval

This research was conducted in compliance with ethical standards and was approved by the ethics committee of the University of Würzburg (local number: 20230203 01).

## Results

### Study cohorts

This study encompassed 23 subject, delineated into distinct cohorts: 11 patients with aSAH, 9 patients serving as *pathological* controls, and 3 patients categorized as *healthy* controls. Among the aSAH cohort, 6 patients exhibited severe aSAH (Hunt and Hess score ≥ 4; mean modified Fisher scale ≥ 3; GCS ≤ 8). The aSAH cohort’s mean age was 64 years (SD 7 years), maintaining a male-to-female ratio of 5:6. The initial mean Glasgow Coma Scale was 8 (SD 4), with a mean Hunt and Hess score of 4 (SD 1) and modified Fisher scale at 3 (SD 1). Eight patients presented with anterior circulation aneurysms, while 3 had a posterior circulation aneurysm. Treatment strategies involved surgical intervention (clipping) for two patients, endovascular procedures (5 coiling and 3 flow diverter) for eight patients, and conservative management for one patient. All aSAH patients received external ventricular drainage upon admission. Notably, none had a pre-existing chronic neurological condition or history of invasive central nervous system treatment. The pathological control cohort encompassed patients with diverse conditions: severe traumatic brain injury (TBI) (*n* = 3), tumors (*n* = 2 carcinoma metastasis and *n* = 1 glioblastoma), intraventricular hemorrhage (IVH) (*n* = 2), and ventriculoperitoneal shunt infection (*n* = 1). The mean age of this sub-cohort was 50 years (SD 15 years), consisting of 7 males and 1 female. affecting the CNS were present among the remaining cases. Seven patients within this group underwent EVD placement, with two receiving a lumbar drain.

### TMEM119-positive cells in aSAH patients

We examined a total of 140 specimens, averaging 6.9 samples per aSAH patient or pathological control (total 20 subject). Among these, 77 cytospin preparations were analyzed in the aSAH patient group, with a higher average of 11 samples per patient. Additionally, 60 cytospin preparations were analyzed in the pathological control group, averaging 6.7 samples per patient. The negative control group underwent analysis with three cytospin preparations, averaging one sample per patient.

In our analysis of 11 aSAH patients, we consistently observed the presence of TMEM119-positive microglial cells.

**Fig. 1:**
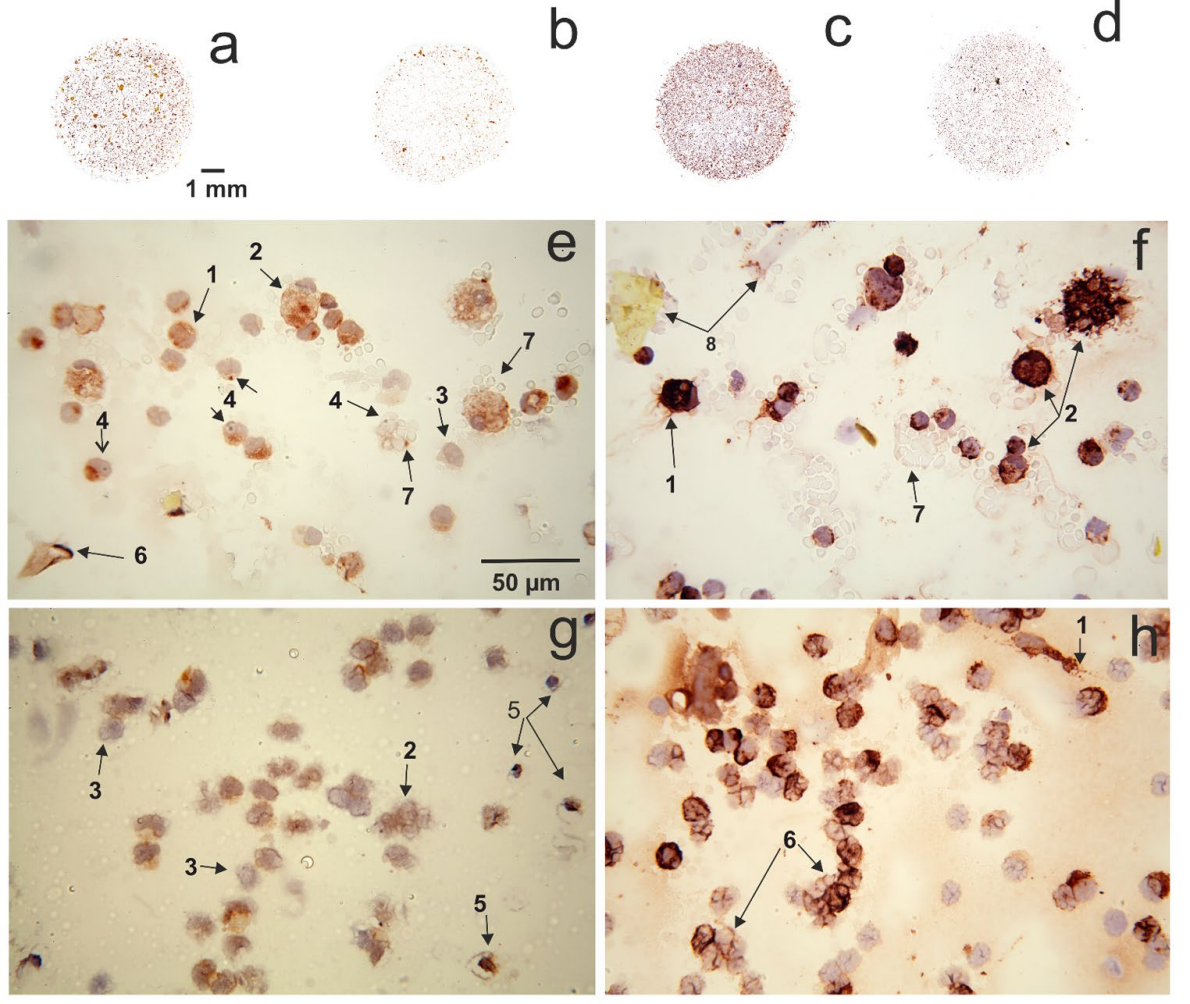
Low-resolution overview of cytospin preparations of aSAH patient group (**a-d**): CD68 (**a**,** c**) and TMEM119 staining (**b**,** d**). The calibration bar in **a** is applicable to figures a-d. (**e-g**) Higher magnification from representative- TMEM119 -(**e**,** g**) and CD68- stained (**f**,** h**) fields.Calibration bar in **e** is applicable to figures f-h. Increased cell-size through phagocytosis (**e1**,**f1**) or syncitial fusion of individual cells (**e2**,** f2**,**g2**). Cells with filopodia (**f1**,** h1**) and erythrophagocytosis (**h6**). Unstained lymphoid cells (**e3**). Intracytoplasmic inclusions bodies in cells with increasing amounts of TMM-positive material (**e4**), degenerate TMEM119-positive cells (**g5**) and rare cells with a unique shape (**e6**). (**e7**,**f7**) accumulated erythrocytes, **f8**) blood clot and probable debris of degenerated tissue

A significant difference in cell density between CD68-positive cells (Fig. 1, a and c) and TMEM119-positive microglial cells (Fig. 1, b and d) was observed. At higher magnification (Fig. 1, e and g), TMEM119-positive microglial cells exhibited an amoeboid shape with a tendency to fuse with one another, forming a syncytium (Fig. 1, e2, g2).Within the cytoplasm of TMEM119-positive cells fine inclusions bodies could be detected (Fig. 1, e4). Individual cells, apparently corresponding to lymphocytes, remain negative for TMEM119 (Fig. 1, e3, g3), as expected. There were also TMEM119-positive microglial cells which appear to degenerate (Fig. 1, g5). A rarely occurring TMEM119-positive cell with a unique shape and two recognizable cell processes could be observed, too (Fig. 1, e6).

**Fig. 2:**
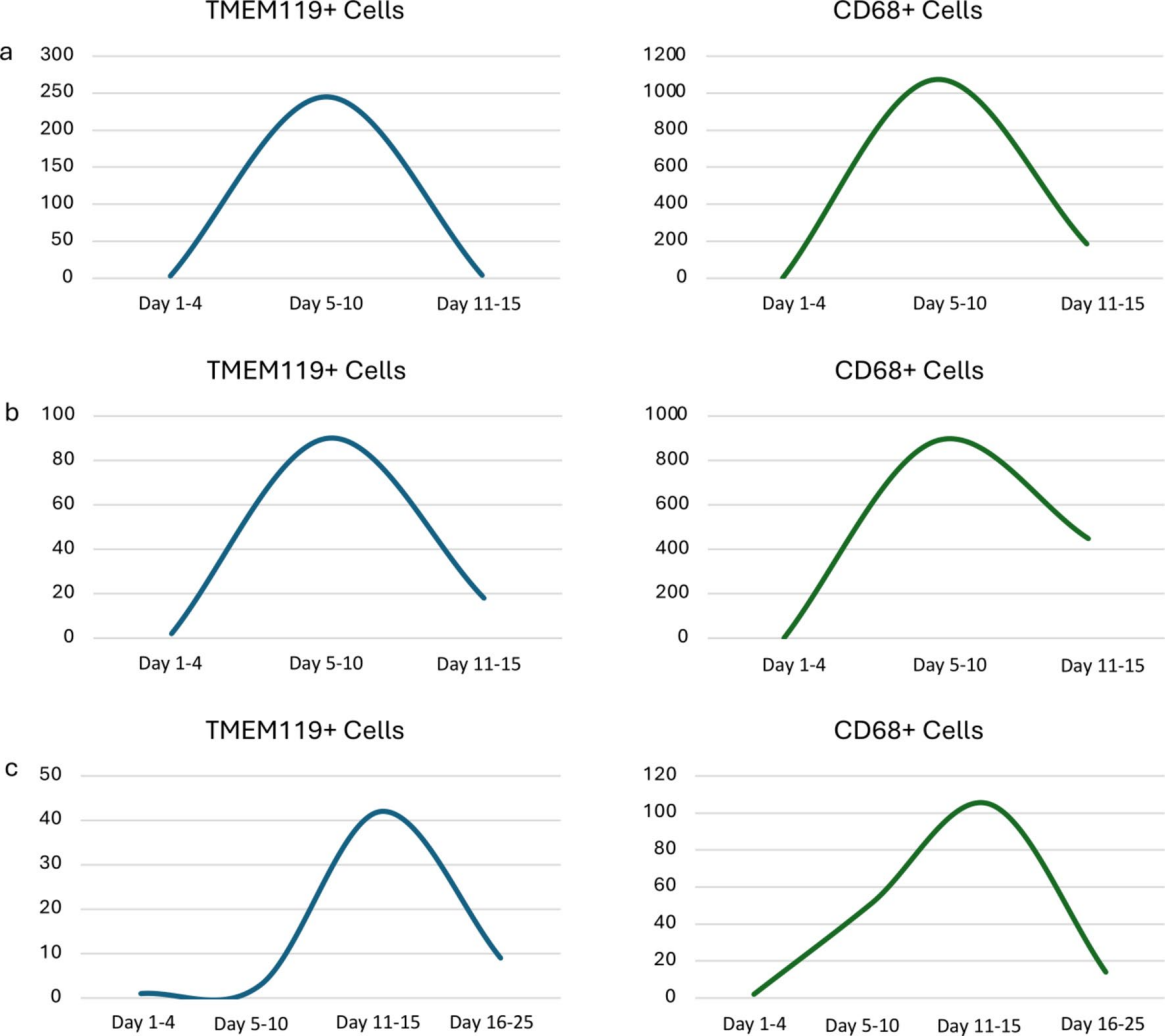
Cell density of cytospin preparations of 3 patients of aSAH group (**a**,** b**,** c**, respectively patient 1, 2, 3). A peak of TMEM119-positive and CD68 during the initial phase until day 10, followed by a gradual decline starting around day 10 post-bleeding could be observed. In each time window was collected a sample of CSF. In case of more sample collected data were averaged

The cytospin preparations are compatible in term of shape, staining intensity and staining specificity with previously published data in our group [27]. We collected patient samples from day 1 until day 26 (in a single case). Remarkably, we detected the presence of microglial cells already in the early days following the onset of bleeding. Some patients displayed these microglial cells in CSF as early as the day after bleeding, while in one case, these cells were still present on day 26. However, and important to note, cells were not present from day 1 in all patients showing a possible patient specific dynamic.

In three patients we were able to collect samples continuously throughout the acute phase. We divided the acute phase in hyperacute (day 1–4), acute (day 5–10) and subacute (day 11–15 and 15–20), and built a graphic to describe the tendences, we collected for those patients one sample per each phase. Even if coming from a small group of patients, we can provide initial data concerning the immunocytochemical presence of microglial cells in CSF of aSAH subjects. In the three patients under continuous observation during the entire acute phase, we noted diverse trends in cell numbers (Fig. 2, a, b,c). There was a distinct peak in microglial cell presence during the initial phase until day 10, followed by a gradual decline starting around day 10 post-bleeding in all 3 patients. All three patients in this small cohort received endovascular treatment and did not undergo any surgical interventions except for external ventricular shunt placement. The average stay in the neurosurgical intensive care unit period for these patients in our facility was 24 days (SD 6 days). All patients in the aSAH group had an EVD inserted on the day of admission. For detailed information on the total cohort, we refer to the table (Table 1a, b).

**Table 1:** Characteristics of all patients (**a**) and pathological controls (**b**) delineated into distinct cohorts of this study

**(a)**			
**aSAH Group**	**Mean (SD)**	**Median**	***N*** **(11)**
Number of samples per patient	7 (4,1)	5	-
Gender (male: female)			5:6
Age at event	64 (7)	65	-
Total days at intensive care unit	20 (7)	21	-
GCS (> 8)	8.3 (4.2)	7	8
Hunt and Hess Score at admission (1–3: 4–5)	3.6 (1.2)	4	3:8
WFNS grade	3.6 (1.6)	4	-
Initial Modified Ranking Scale	3.4 (1.9)	4	-
Modified Fisher grade at admission	3.2 (0.9)	3	-
Aneurysm location (anterior circulation: posterior circulation)	-	-	9:2
Total days with EVD	17 (6)	16	
CSF drainage (1 EVD: 2 EVD: Lumbar Drainage)	-	-	11:0:0
EVD revision	-	-	2
Ventriculitis	-	-	1
Intracerebral hemorrhage (at admission: secondary)	-	-	0
Surgical treatment for intracerebral hemorrhage	-	-	0
Decompressive craniectomy	-	-	0
Initial treatment for aneurysm (endovascular: surgical: conservative)	-	-	8:2:1
Additional surgical treatment to endovascular treatment	-	-	1
Post-Operative / post-Angiography rebleeding	-	-	1
Vasospasm	-	-	6
Patient with transcranial doppler detected vasospasm	-	-	6
Patient with angiographic confirmed vasospasm	-	-	6
Patient with perfusion CT detected vasospasm	-	-	6
Patient with delayed cerebral ischemia	-	-	3
Patient who needed an angiographic intervention for vasospasm	-	-	6
Number of angiographic intervention for vasospasm per patient	2.6 (2.7)	1	-
**(b)**			
**Control group**	**Mean (SD)**	**Median**	**N (9)**
Number of samples per patient	6,7 (5,5)	5	
Gender (male: female)	-	-	7:2
Age at event	50 (15)	60	-
Total days at intensive care unit	32 (19)	24	-
GCS (> 8)	11 (5)	14	4
Initial Modified Ranking Scale	2.1 (SD 2.5)	0	-
CSF Drainage (1 EVD: 2 EVD: lumbar drainage)	-	-	6:1:2
Total days with EVD	16 (13)	17	-
EVD revision	-	-	2
Ventriculitis	-	-	2
Intracerebral hemorrhage (At admission: secondary)	-	-	4:3
Surgical treatment for intracerebral hemorrhage	-	-	4
Decompressive craniectomy	-	-	1
Overall surgical treatment	-	-	7
Glasgow Outcome Score at discharge (1–3: 4–5)	2.7 (1.2)	2	7:2
Modified Ranking Scale at discharge (1–3: 4–6)	4.3 (1.5)	5	7:2

### TMEM119 positive cells in control patients

Regarding the pathological control patients, we observed a significant variation in the number of cells among different individuals. The present study lacks the requisite power to describe a potential difference in cell quantity across the various pathological conditions under investigation. Nevertheless, it is noteworthy that TMEM119-positive microglial cells were consistently observed in all pathological states examined, including TBI, tumor, infection, and spontaneous IVH.

It is noteworthy that six of the nine patients observed had undergone one or more brain surgeries. Two of them had been the subject of a decompression surgery. Only one patient had not undergone any intracranial surgery. With regard to the healthy controls, no TMEM119-labeled cells or other cells were observed in the analyzed CSF samples.

### CD68 positive cells in SAH patients

To compare the density of cells positive for TMEM119 with the myeloid population present in cerebrospinal fluid (CSF) following an aneurysmal subarachnoid hemorrhage (aSAH), we performed CD68 staining in the three patients with serial sampling. The purpose of this staining was to assess the presence of macrophages, including the presence of microglial cells and macrophages derived from the periphery.In comparison to cytospin preparations of TMEM119-positive profiles the cytospin preparations showed a higher cell density of CD68-positive cells shown in Fig. 1a, c. There is a tendency to cell increase in size through phagocytosis (Fig. 1, f1). Some of the CD68-positive cells showed multiple fine cell processes (Fig. 1, f1, h1). Erythrophagocytosis was also present (Fig. 1, h6). In addition to numerous cellular elements, larger irregularly shaped profiles can be seen at low magnification in Fig. These profiles most likely represent blood clots or pieces of degenerated neuronal tissue from superficial cortical regions (Fig. 1, f8). They are preferentially targeted by CD68-positive cells (Fig. 1, f8). Erythrocytes (Fig. 1, e, f7) are taken up by both TMEM119- and CD68-positive cells. The shape and intracellular distribution of TMEM119 and CD68 antibodies give a honeycomb appearance to CD68-positive cells (Fig. 1, h6) and a more vesicular appearance to TMEM119-positive cells (Fig. 1, g2).

The typical pattern of inflammation described after aSAH [7, 9], is following the pattern of total CD68 positive cells displayed in Fig. 2. In this group of patients, the trend of CD68 and TMEM119 positive cells matched, and the profiles looked quite similar for the three patients, respectively. Nevertheless, we can only offer a descriptive non-quantitative trend because of the small study group.

## Discussion

In our study, we consistently identified TMEM119-positive microglial cells in CSF after aSAH. To our knowledge, we are the first to describe the immunocytochemical presence of TMEM119-positive microglial cells in CSF after aSAH. Furthermore we confirmed our study on post-mortem specimens (Bohnert et al. 2022; Bohnert et al. 2020). We observed a heterogeneous distribution of microglial cells in the CSF, with some patients showing such cells as early as day 1 post-bleeding and no disappearance of these cells throughout the acute phase. In the three patients monitored continuously during the acute phase, we noted varying trends in microglial cell numbers, with a peak in the initial phase and a gradual decline after day 10. These findings offer insights into the presence and distribution of microglial cells in CSF of aSAH patients.

In recent years, the understanding of pathology in brain damage after aSAH has changed radically. Currently, the mechanisms involving the microenvironment have gained importance (Geraghty and Testai 2017; Osgood 2021; Plesnila 2022; Geraghty et al. 2019) and the effect of macrovasospasm has been reconsidered. There is a great ongoing interest in understanding neuroinflammatory processes that manifest in both the acute and chronic phases within the CNS after aSAH as much as the inflammatory state that affect the entire body systemically (Mohme et al. 2020; Roa et al. 2020; Liao et al. 2023; Weiland et al. 2021; Geraghty et al. 2019; Ridwan et al. 2021). The pathological phenomena observed in the acute phase such as increase in intracranial pressure, reduction in cerebral blood flow and, consequently, a reduction in cerebral perfusion lead to the activation of a cascade of events that continue into the subacute phase, called early brain injury (Osgood 2021). In this phase, a series of inflammatory mechanisms are activated in the brain tissue. The different neuroinflammatory mechanisms that are activated appear to sustain brain injury (Group et al. 2019; Weiland et al. 2021). Furthermore, these inflammatory phenomena exhibit prolonged activity in animal models of SAH, extending well beyond the traditionally defined chronic phase (Patsouris et al. 2023). Inflammatory responses following aSAH within the brain can be indirectly observed through CSF analysis, routinely monitored in the majority of aSAH patients with external ventricular drainage but neglected as source of immunocytochemistry in daily practice until now. Limited data from smaller cohorts have employed detailed immune reaction profiling using flow cytometry. Some correlations have been noted between these inflammatory phenomena and the occurrence of DCI (Mohme et al. 2020; Roa et al. 2020). An aspect that has received limited attention in human studies is the function of microglia due to the technical challenges involved. Microglia, residing in brain tissue that is largely inaccessible in most cases until death, have only recently been observed in the CSF of patients (Roa et al. 2020). The earliest documented instances, to our knowledge, is detailed in the study by Roa et al. (Roa et al. 2020), who, using flow cytometry, described CD11high and CD45mid cells in the CSF.

Microglia play a pivotal role in neuroinflammatory responses in CNS diseases, and their activation can be regarded as a marker for the inflammatory reactions that occur following brain injuries (Prinz et al. 2019; Masuda et al. 2019; Franco and Fernández-Suárez 2015). The multifaceted tasks and roles executed by microglia in various disease states represents a paramount challenge in contemporary neuroinflammation research. In the context of aneurysmal subarachnoid hemorrhage, microglia play an essential role in the development of secondary damage (Schneider et al. 2015). Numerous studies in the literature have detailed how the modulation of microglia through different pathways can significantly impact the outcomes after aSAH (Chen et al. 2022). It is well-established that the activation state of microglia following aSAH is predominantly proinflammatory, highlighting their central involvement in the pathophysiological processes associated with this condition (Zeng et al. 2020).

Recent advances have significantly improved our understanding of brain anatomy and its interactions with the immune system. Emerging evidence reveals the presence of direct vascular channels between the skull bone marrow and the brain surface (Herrison et al. 2018), and direct connection between the brain and the dura mater (Smyth et al. 2024), facilitating the migration of myeloid cells and enabling local immune responses within the brain. Additionally, the discovery of a fourth meningeal layer, the subarachnoid lymphatic-like membrane (SLYM), has highlighted the crucial role of those layers in compartmentalizing the subarachnoid space, regulating CSF flow, and hosting immune cells (Mollgard et al. 2023).

Moreover, the advanced understanding of the function of the glymphatic system, has shown its role to be integral part of the complex interaction system between the brain, its protective membranes, and the immune system (Hablitz and Nedergaard 2021a). These findings underscore the complex interactions between the brain and the immune system also outside the proper brain tissue. This interaction is possibly more complex than hitherto known and can also occur through previously unknown pathways.

Building on this background, our study aimed to explore the presence of microglial cells in the CSF using a robust marker, TMEM119, and an alternative technique to flow cytometry, namely immunocytochemistry. TMEM119 has been a widely accepted and reliable pan microglial marker since its introduction in 2015 (Prinz et al. 2019; Satoh et al. 2016; Ruan and Elyaman 2022; Bohnert et al. 2020; Paolicell et al. 2022), despite recent critiques (Vankriekelsvenne et al. 2022). Our research, in conjunction with the findings by Roa et al. (Roa et al. 2020), confirms the presence of microglial cells in the CSF of living patients.

Notably, our group had previously identified microglial cells also in the post mortem CSF of individuals with intracranial pathology (Bohnert et al. 2022), suggesting that microglial cells may migrate into the CSF in different neuropathological settings or during dying. These findings were corroborated in the here given study, where we observed the presence of microglial cells not only in aSAH patients but also in individuals with various conditions such as trauma, tumors, infections and intraventricular hemorrhage, but - to be highlighted - not in healthy controls at all.

Building upon our prior research (Bohnert et al. 2022), we have hypothesized a potential mechanism explaining this migration, suggesting a possible link between the presence of microglial cells in the cortical brain cortex and their appearance in the CSF. While the precise reasons for microglial migration within the CSF and its mechanism remain elusive, our study lays the groundwork for further exploration. Hypothetically, microglia might invade the CSF to execute their functions at sites distal from the initial injury (Zeng et al. 2020). Existing literature provides instances of microglia adopting an amoeboid form and migrating centrifugally from the primary site of injury, supporting the proposition that microglial cells may undertake a journey via CSF transport to perform their roles in remote regions e.g. waste removal independently by the CNS glymphatic pathway (Hablitz and Nedergaard 2021b; Iliff et al. 2012; Benveniste et al. 2017).

## Limitations

It is essential to note that our study was primarily designed to be a preliminary exploration, and a direct, immunocytochemical proof of the presence of microglia in the CSF, and therefore has neither the design nor the statistical power to correlate clinical outcomes with the presence of microglia. Therefore, patients included are heterogenous. Moreover, in some of the pathological control subjects and in all the healthy control subjects CSF was collected via lumbar puncture/drainage which could potentially lead to differences in the CSF composition.

Aware of these limitations, our work stands as a foundation for future studies on the presence of microglial cells in various neuropathological disorders.

## Conclusion

Our study demonstrates the presence of TMEM119-positive microglial cells in CSF of aSAH patients and other neuropathological conditions, but not in healthy controls, suggesting potential migration into CSF in pathological conditions to carry out functions away from the primary damaged site. In conclusion, this preliminary study opens the door to further research on the role of microglia and neuroinflammation in the aftermath of aSAH. Our findings, provide valuable insights and offer avenues for understanding the pathophysiology of this condition. Individual differences, different clinical courses but also methodological differences could explain the heterogeneity of our results and require further investigation. The presence of TMEM119-positive microglial cells in the CSF may serve as an early predictor of inflammatory-related central nervous system damage, holding promise for improved diagnostics and potential therapeutic interventions in the future.

## Abbreviations

aSAH: Aneurysmal subarachnoid hemorrhage
CSF: Cerebrospinal fluid
DCI: Delayed cerebral ischemia
BBB: Blood-brain barrier
CNS: Central nervous system
EVD: External ventricular drainage
HE: Hematoxylin-eosin
GCS: Glasgow Coma Scale
TBI: Traumatic brain injury
IVH: Intraventricular hemorrhage
